# Examination of “Pain-Killers”

**Published:** 1877-01-20

**Authors:** 


### EXAMINATION OF "PAIN-KILLERS."

Jos. J. Pierron, Ph. C., in the *Peninsular Journal of Medicine*, gives the following abstract of a report on file in the Michigan University School of Pharmacy:

*Perry Davis' Pain Killer.* In a bottle sold for a dollar:

Spirit of camphor, about......fl.oz.ij;
Tinct. of capsicum, about.....fl.oz.i;
Guaiac................................oz.ss;
Alcohol...............................fl.oz.iij;
Myrrh and color.

*Radway's Ready Relief.* In a half-dollar bottle:

Soap liniment, about.........fl.oz.iss;
Tinct. of capsicum..............fl.oz.ss;
Water of ammonia.............fl.oz.ss;
Alcohol...........................fl.oz.ss.

*Flagg's Relief.* In a bottle sold for half a dollar:

Oil of cloves, about.........fl.dr.i;
Oil of sassafras.................fl.dr.ij;
Spirit of camphor,.............fl.oz.iss.

*Chamberlain's Relief.* In a bottle sold for thirty-five cents (approximately):

Tinct. of capsicum.............fl.oz.i;

Spirit of camphor,..............fl.oz.$\frac{3}{4}$;

Guaiac,..........................fl.oz.$\frac{1}{4}$;

Color tincture, to make two fluid ounces.

*Hamlin's Wizard Oil.* In a bottle sold for a dollar there are (in approximate proportion):

Spirit of camphor............fl.oz.i;
Spirit of ammonia............fl.oz.ss;
Oil of sassafras,...............fl.oz.ss;
Oil of cloves,....................fl.dr.ij;
Chloroform.........................fl.oz.ss;
Oil of turpentine...............fl.oz.ss;
Alcohol, to make about five fluid ounces.

*Kellogg's Red Drops.* A bottle, sold for half a dollar, contains (in approximate quantities):

Spirit of camphor,............fl.oz.ij;
Spirit of origanam............fl.oz.$\frac{1}{4}$;
Oil of sassafras................fl.oz.$\frac{1}{4}$;
Oil of turpentine...............fl.oz.ss;
Color tincture, to make three and a fourth fluid ounces.

—*Louisville Medical News.*

